# Uncovering Hidden Gluten Exposure in Celiac Patients: A Case Study in Family-Based Management and the Role of Point-of-Care Urine Testing and Psychological Assessment

**DOI:** 10.3390/ijms26115135

**Published:** 2025-05-27

**Authors:** Ángela Ruiz-Carnicer, Cristóbal Coronel-Rodríguez, María Cinta Guisado-Rasco, Isabel Comino, Carolina Sousa, Verónica Segura

**Affiliations:** 1 Department of Microbiology and Parasitology, Faculty of Pharmacy, University of Seville, 41012 Seville, Spain; acarnicer@us.es (Á.R.-C.); icomino@us.es (I.C.); csoumar@us.es (C.S.); 2 Health Center Amante Laffon, 41010 Seville, Spain; cristobalcoronel@gmail.com (C.C.-R.); cintaguisado@gmail.com (M.C.G.-R.)

**Keywords:** celiac disease, gluten immunogenic peptides, point-of-care testing, primary care, psychological assessment

## Abstract

Celiac disease (CD) is a chronic immune-mediated enteropathy that requires strict adherence to a gluten-free diet (GFD) to prevent intestinal damage. Traditional methods for monitoring GFD adherence, such as serology and dietary assessments, often poorly correlate with histological findings and typically involve a waiting period before results are available, limiting their usefulness for immediate clinical decision-making. This cross-sectional case study reports on a 45-year-old mother and her 11-year-old twin daughters, all diagnosed with CD and following a GFD for over two years. Despite being asymptomatic and showing negative anti-tTG serology, the mother continued to present Marsh 1 histological lesions, suggesting ongoing subclinical inflammation. Point-of-care testing (POCT) for gluten immunogenic peptides (GIP) in urine revealed positive results for all three individuals, indicating recent gluten exposure despite reported dietary adherence. A follow-up GIP test after dietary review and reinforcement yielded negative results, confirming improved adherence. Additionally, a psychological assessment using the Hospital Anxiety and Depression Scale (HADS) revealed anxiety symptoms in the mother and one of the daughters, which may have influenced adherence to the GFD. These findings underscore the clinical value of urinary GIP POCT as a rapid, non-invasive tool for detecting hidden gluten exposure, even when traditional monitoring appears normal. Integrating GIP testing and psychological screening into routine clinical practice may enhance management and support timely, personalized interventions in patients with CD.

## 1. Introduction

Celiac disease (CD) is a systemic disorder characterized by chronic inflammatory enteropathy of the small intestine due to an inappropriate immune response to gluten in genetically predisposed individuals [1,2]. It is estimated to affect approximately 1.4% of the global population [3], though prevalence varies by age and region. CD can present with gastrointestinal symptoms, such as chronic diarrhea and abdominal pain, or extraintestinal manifestations, including oral, skin, neurological, joint, endocrine, and hematological alterations, among others. It may also remain asymptomatic. Accurate diagnosis requires a combination of clinical, serological, and histopathological evaluations [4].

Currently, the only available treatment for CD is strict adherence to a lifelong gluten-free diet (GFD) [4]. Strict adherence to the GFD is essential to alleviate symptoms, prevent nutritional deficiencies, and improve the quality of life for these patients, while also reducing short- to medium-term complications and healthcare costs. However, numerous studies suggest that both voluntary and involuntary dietary transgressions are relatively common [5]. Major reasons for non-adherence include social circumstances, the high cost of special dietary products, and technological challenges in ensuring gluten-free status in manufactured foods [5]. In addition to these factors, following a GFD can lead to issues such as anxiety, depression, or stress, which negatively impact dietary adherence. Therefore, the incorporation of validated psychological questionnaires into routine follow-up may provide valuable insights into the mental well-being of patients and help identify those at greater risk of non-adherence with the GFD [6,7,8]. Early identification and psychological support can play a critical role in improving long-term adherence and overall health outcomes.

Nonetheless, one of the most significant challenges remains the lack of accurate markers for monitoring dietary adherence, as symptoms, serology, and adherence questionnaires have proven ineffective for this purpose [9]. Serology (such as tissue transglutaminase (anti-tTG) and deamidated gliadin (anti-DGP) antibodies) and dietary assessment are commonly used in clinical care but have limited correlation with persistent histological findings, while patients’ perception of gluten exposure is often inaccurate [10,11]. In addition, serological testing typically involves a waiting period before results are available, reducing its utility for immediate clinical decision-making.

Recently, new point-of-care testing (POCT) methodologies have been developed that are rapid, non-invasive, precise, and reliable for monitoring gluten exposure in patients with CD adhering to a GFD [11,12,13,14,15,16,17]. These methodologies are based on the detection of gluten immunogenic peptides (GIP) in urine samples, which are fragments of gluten that remain intact during digestion and are excreted shortly after gluten consumption. GIP testing has proven to be a sensitive and clinically validated biomarker capable of detecting recent gluten exposure and demonstrating the absence of intestinal damage in patients with CD on a GFD [11,14]. This avoids the need for repeated invasive biopsies during the follow-up [4,11].

In this study, the potential role of POCT-based GIP testing in primary care settings is examined as a rapid and non-invasive method for monitoring dietary adherence in patients with CD. The novelty of this brief report lies in the implementation of GIP testing directly within a primary care environment, rather than in a specialized or hospital-based setting. This approach enables more timely and accessible assessments of gluten exposures, supports more consistent and personalized follow-up, and may ultimately improve long-term dietary adherence while reducing the risk of complications. Additionally, this study integrates a psychological evaluation using the Hospital Anxiety Scale (HAS) to explore the emotional and cognitive impact of dietary adherence and POCT on patients with CD.

## 2. Results

### 2.1. Patients

The study population consisted of three individuals diagnosed with CD three years prior to the follow-up visit: a 45-year-old mother and her 11-year-old twin daughters. At the time of diagnosis, the mother and one of the twins had Marsh 3b lesions, while the other twin had Marsh 3c lesions. All individuals had positive anti-tTG serology at diagnosis: the mother with a value of 120 U/mL, one twin with 21 U/mL, and the other twin with 17 U/mL. At the time of the CD follow-up visit, all participants had been on a GFD for over 24 months.

### 2.2. Clinical Follow-Up and Monitoring During CD Follow-Up Visit

After diagnosis, the family strictly followed the GFD. During a primary care follow-up visit, a comprehensive clinical evaluation was performed.

#### 2.2.1. Biochemical Evaluation

Comprehensive laboratory assessments demonstrated normal results for complete blood count, iron metabolism, thyroid profile, and vitamin B12 levels in the mother and both twins. Despite these normal findings, certain deficiencies were observed in essential micronutrients. Specifically, the mother exhibited vitamin D deficiency (<20 ng/mL), with a recorded level of 16 ng/mL, while the twins displayed insufficient vitamin D levels (20–29 ng/mL), measuring 24.7 ng/mL and 25.1 ng/mL, respectively. Moreover, one of the twins presented with low folic acid levels (2.6 ng/mL), warranting closer attention. These biochemical anomalies, alongside the otherwise normal laboratory parameters, underscore the importance of ongoing nutritional monitoring in patients with CD on a GFD.

#### 2.2.2. CD Antibodies and Duodenal Histology

During the primary care follow-up visit, it was found that the mother’s anti-tTG serology was negative (0.5 U/mL), and both twins also showed negative anti-tTG results (3.1 U/mL and 2.1 U/mL, respectively). Therefore, after more than 24 months of adherence to a GFD, the concentrations of serum anti-tTG antibodies had declined since the diagnosis. However, despite the negative serological results, the mother’s follow-up duodenal biopsy revealed persistent lymphocytic infiltration, indicative of Marsh 1 lesions, suggesting ongoing subclinical inflammation. In contrast, no follow-up biopsies were performed on the twin daughters, in accordance with current ESPGHAN guidelines, which do not recommend routine biopsies in pediatric patients with confirmed CD who show good clinical and serological response to the GFD.

#### 2.2.3. Celiac Dietary Adherence Test (CDAT)

The patients with CD were asked to fill out the CDAT to assess their GFD adherence. According to the CDAT, the mother scored 11 points, while her twin daughters each scored 10 points. These scores reflect a strong adherence to the GFD, suggesting that all family members have been effectively following the dietary recommendations. The consistency in their adherence to the GFD was further supported by their self-reported confidence in maintaining strict dietary control, as well as their efforts in meal planning and careful avoidance of gluten-containing foods.

#### 2.2.4. Symptomatology

The family reported being asymptomatic according to the symptom questionnaire completed during the visit and expressed confidence in their adherence to the GFD. They indicated that the adoption of the GFD had resulted in a significant improvement in their quality of life, with no gastrointestinal discomfort or other related symptoms. The family also mentioned employing various strategies, such as thorough label reading and home meal preparation, to ensure strict adherence.

#### 2.2.5. Psychological Assessment of Adherence to the GFD

Psychological well-being plays a pivotal role in successful adherence to a GFD. As part of the comprehensive follow-up, a psychological assessment was conducted to evaluate the impact of mental health on dietary adherence. The results revealed that both the mother and one of the twin daughters had anxiety problems, with each scoring 12 points on the anxiety scale. This suggests that anxiety may influence their ability to maintain the GFD. In contrast, the second twin demonstrated a significantly lower score of 5 points, indicating no signs of anxiety and suggesting a better psychological alignment with dietary adherence. These findings underscore the importance of considering psychological factors when assessing and supporting patients with CD in managing their GFD.

#### 2.2.6. POCT-Based GIP Determination in Primary Care

Considering the discordant results between negative serology and persistent histological lesions in the mother, a POCT was utilized to assess gluten exposure in all three family members during their primary care follow-up. The POCT revealed GIP positive in the urine of all family members, indicating recent gluten ingestion despite their self-reported adherence to a strict GFD. These positive results prompted a thorough investigation into potential sources of gluten exposure, which were discussed with the family. Following this consultation, the patients were provided with tailored guidance to further refine their GFD practices and eliminate potential sources of inadvertent gluten contamination. After a period of follow-up with the new recommendations, the GIP test was repeated and yielded a negative result, suggesting a successful resolution of the issue and confirming the family’s improved dietary adherence.

## 3. Discussion

The detection of GIP in feces and urine through immunoassays (ELISA and LFIA) has emerged as a non-invasive method for assessing adherence to a GFD in patients with CD [10,11,12,13,14,15,16,17]. Unlike traditional tools that only measure the consequences of gluten ingestion, GIP detection allows identification of the timing, sources, and quantities of gluten consumed [11,14,18]. These immunoassays have been validated in various clinical studies, revealing limitations of clinical symptoms, serological tests, and dietary questionnaires in detecting gluten exposure and dietary transgressions [10,11,12,13,14,15,16,17,18,19].

Previous studies have shown that while dietary questionnaires and serology detected gluten exposure in only 18–30% of patients, GIP detection uncovered exposure in 70% of patients who were unaware of ingesting gluten [19].

Fecal GIP testing, especially through LFIA, offers a simpler tool for detecting gluten consumption, while ELISA provides greater sensitivity and quantification [10,19]. This is particularly valuable in pediatric populations, where gluten exposure has been detected in 8–35% of patients considered adherent by dietary questionnaires [19]. Moreover, GIP levels have been associated with mucosal damage; in one study, 39% of patients with positive GIP results showed histological evidence of intestinal injury, compared to only 3% of those with negative GIP results [15]. GIP urinary testing has also demonstrated high sensitivity in identifying dietary transgressions, making it a promising tool for long-term follow-up.

Despite the high sensitivity of GIP testing, conventional monitoring tools like serological tests and symptom evaluations often fail to capture intermittent gluten exposure, leading to intestinal damage and long-term complications [11]. Our findings reinforce the need for continuous and objective monitoring in CD. In this study, GIP were detected in the urine of a mother and her twin daughters, all of whom reported strict adherence to the GFD. This discrepancy highlights the inadequacy of traditional assessment tools and underscores the importance of incorporating more accurate methods for monitoring gluten intake. Serology and dietary assessments are commonly used in clinical care but often do not align with persistent histological findings, and patients’ perceptions of gluten exposure can be inaccurate. The positive GIP results led to a reassessment of the patients’ dietary practices and a discussion about potential unnoticed sources of gluten exposure.

Moreover, an aspect that is becoming increasingly important is the psychological factor of adherence to a GFD in patients with CD. Psychological well-being plays a crucial role in maintaining strict adherence to the GFD, yet it is often overlooked in routine clinical follow-up [20]. Anxiety and other psychological factors can negatively affect adherence to the diet, leading to inadvertent gluten exposure. These issues can reduce motivation and coping abilities, making it harder for patients to maintain dietary restrictions. Integrating psychological support into CD management could help address these challenges. By including mental health assessments and interventions as part of routine care, healthcare providers can better identify patients at risk for poor adherence. This holistic approach could improve both dietary compliance and overall clinical outcomes, offering a more comprehensive strategy for long-term disease management.

In addition to psychological and behavioral factors, nutritional monitoring is also essential in the follow-up of patients with CD. Suboptimal adherence to a GFD can lead to persistently low levels of key micronutrients such as vitamin D, folic acid, iron, and vitamin B12, despite apparent clinical and serological remission [21,22]. These deficiencies may reflect ongoing intestinal inflammation or malabsorption due to inadvertent gluten exposure. One study revealed significantly higher micronutrient deficiencies in patients with CD compared to healthy controls, highlighting the importance of systematic nutritional assessment and multidisciplinary management to address these deficiencies and minimize their potential negative impact on overall health. Therefore, routine assessment of micronutrient status should be considered an important component of comprehensive CD management, helping to identify patients who may require further dietary evaluation or support [23].

Building on this, integrating POCT for GIP into primary care offers significant advantages. POCT provides rapid and non-invasive results, enabling healthcare providers to quickly identify dietary transgressions and adjust dietary recommendations as needed. In this study, POCT-based GIP testing led to a reassessment of the patients’ dietary practices, demonstrating its effectiveness in detecting hidden gluten exposure. Regular GIP monitoring can optimize CD management, particularly in primary care, by preventing unnecessary referrals and ensuring early intervention.

Furthermore, incorporating POCT-based GIP testing into routine clinical practice can address broader challenges for patients with CD, such as difficulties interpreting food labeling, social pressures, and the financial burden of maintaining a strict GFD. While adherence to the diet is essential for symptom control, mucosal healing, and the prevention of long-term complications, unintentional gluten exposure remains frequent, even among patients who believe they are adhering to the diet. The use of POCT-based GIP in primary care offers a practical, non-invasive, and timely solution for detecting dietary transgressions, facilitating early intervention without overburdening specialized services. This strategy supports a more proactive and personalized model of care, aligning with the principles of patient-centered healthcare. By improving the accuracy of dietary monitoring and empowering both patients and clinicians, POCT-based GIP testing in primary care settings may significantly enhance long-term disease management and overall quality of life for individuals with CD.

## 4. Materials and Methods

### 4.1. Study Design and Population

This cross-sectional follow-up study was conducted at the Amante Laffón Primary Care Center (Seville, Spain). It involved a woman and her twin daughters, all of whom had been diagnosed with CD three years earlier. The participants attended a routine follow-up visit during which a clinical evaluation was performed, including a review of serological markers and laboratory parameters. The study protocol was approved by the local ethics committee, and written informed consent was obtained from all participants ≥ 12 years old, or from the parents or legal guardians in the case of children < 12 years old.

### 4.2. Urine and Blood Collection

The subjects were instructed to collect a 50 to 100 mL sample of urine in a sealed container and were provided with specific instructions to prevent contamination with gluten during sample collection.

Blood samples were collected in order to obtain plasma that was stored at −80 °C until the analysis. The investigators who performed the urine and serum analyses were blinded to the patients’ GFD status at the time of sample collection.

### 4.3. CD Antibodies 

The concentrations of anti-tTG IgA and anti-gliadin (AGA) IgA antibodies (anti-tTG or AGA IgG in IgA-deficient patients) were determined by ELISAs using the EliATM Celikey^®^ IgA/IgG and EliATM GliadinDP IgA/IgG kits, respectively, in accordance with the manufacturer’s protocol (Phadia Laboratory Systems, Thermo Fisher Scientific–ImmunoDiagnostics Division Uppsala, Uppsala County, Sweden). The manufacturer-recommended cutoff of >10 U/mL was used.

### 4.4. Duodenal Histology

At least four endoscopic biopsies of the distal duodenum and two biopsies of the duodenal bulb were performed. Intraepithelial lymphocytes were quantified by immunohistochemical examination using an automated platform, the Ventana Bench-Mark ULTRA (Roche Holding AG, Basel, Basel-Stadt, Switzerland), and CD3 monoclonal antibody concentrations (Roche Holding AG). Mucosal specimens were independently graded according to the Marsh–Oberhuber classification [24,25]. Histological lesions without atrophy observed during follow-up were categorized as Marsh 0–I, while those with villous atrophy were classified as Marsh II–III. Patients with Marsh II lesions were included in the villous atrophy group, as it has been reported in the literature that this degree of histological damage is associated with a high probability of underdiagnosis [26,27].

### 4.5. Celiac Dietary Adherence Test (CDAT)

Adherence to the GFD was evaluated using the Spanish translation of the CDAT with additive scores of 7–35, wherein scores < 13 indicate excellent or good adherence to the diet, whereas scores > 17 reflect fair or poor adherence [28].

### 4.6. Symptoms Questionnaire

All participants were administered a structured questionnaire designed to assess the presence or absence of symptoms related to CD. The questionnaire included closed-ended questions addressing gastrointestinal symptoms (such as abdominal pain, diarrhea, bloating, and nausea) and extraintestinal symptoms (such as fatigue, headaches, mood disturbances, or dermatological issues). Participants indicated whether they had experienced any of these symptoms in the weeks preceding the assessment. This tool allowed for the classification of subjects as symptomatic or asymptomatic at the time of follow-up.

### 4.7. The Hospital Anxiety Scale (HAS)

HAS is a questionnaire made up of 11 items with a 4-point Likert-like scale, where 0 is the lowest score and 3 is the highest one; it detects cognition-related anxiety symptoms (odd and even items, respectively) in the past week. Adding both subscales would provide an overall emotional distress score. In general, a higher score indicated a greater emotional involvement (higher levels of anxiety). Scores of 0–6 indicate a normal absence of anxiety, 7–9 a probable case of anxiety, and >9 clinical problems of anxiety [5].

### 4.8. POCT-Based GIP Determination in Urine Samples

**Figure 1:**
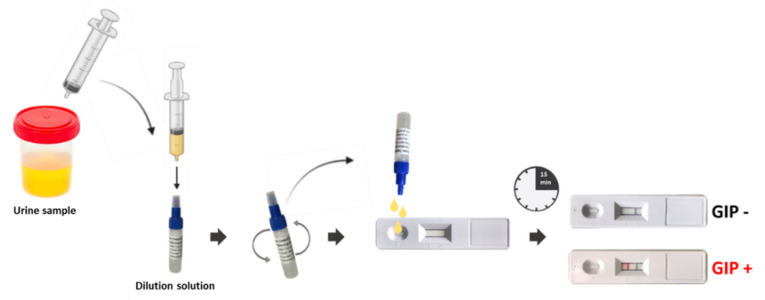
Workflow for POCT-based determination of GIP in urine samples in primary care settings. A positive result is indicated by a red line in the test area, while the absence of this line indicates no GIP (negative result). Additionally, a green control line appears if the test is performed correctly, regardless of the result. GIP, gluten immunogenic peptides; POCT, point-of-care testing.

On the day of the medical consultation, a urine sample was unexpectedly requested from the patients, without prior notice or their knowledge. The GlutenDetect Urine test (Biomedal S.L., Seville, Andalusia, Spain), based on lateral flow devices with monoclonal antibodies (G12 and A1), was used to measure GIP levels. This test provides results in under 15 min (Figure 1). A positive result indicates recent gluten ingestion.

## 5. Conclusions

This study highlights the importance of ongoing monitoring in CD to detect occasional gluten exposures, even in asymptomatic patients. POCT-based GIP detection in urine, performed in the primary care setting, revealed hidden gluten consumption in patients who reported strict adherence to a GFD, despite normal serology and the absence of obvious symptoms. By integrating POCT into primary care, healthcare providers can rapidly detect gluten exposure, enabling prompt interventions without the need to wait for results from other tests. This approach enhances long-term disease management, improves patient outcomes, and provides a more efficient and proactive strategy for managing CD. Additionally, psychological factors, such as anxiety, may influence dietary adherence, underscoring the importance of addressing mental health when managing CD.

## Data Availability

The data presented in this study are available on request from the corresponding author.

## Abbreviations

The following abbreviations are used in this manuscript:

AGAs	Anti-gliadin antibodies
Anti-DGP	Anti-deamidated gliadin antibodies
anti-tTG	Tissue transglutaminase antibodies
CD	Celiac disease
CDAT	Celiac dietary adherence test
GIP	Gluten immunogenic peptides
GFD	Gluten-free diet
HAS	Hospital Anxiety Scale
POCT	Point-of-care testing
